# Study protocol for evaluating the implementation and effectiveness of an emergency department longitudinal patient monitoring system using a mixed-methods approach

**DOI:** 10.1186/s12913-017-2014-9

**Published:** 2017-01-23

**Authors:** Marie Ward, Eilish McAuliffe, Abel Wakai, Una Geary, John Browne, Conor Deasy, Michael Schull, Fiona Boland, Fiona McDaid, Eoin Coughlan, Ronan O’Sullivan

**Affiliations:** 1 School of Nursing, Midwifery and Health Systems, College of Health Sciences, University College Dublin, Belfield, Dublin 4, Ireland; 2 Emergency Care Research Unit (ECRU), Division of Population Health Sciences (PHS), Royal College of Surgeons in Ireland (RCSI), Dublin 2, Ireland; 3 Department of Emergency Medicine, Beaumont Hospital, Dublin 9, Ireland; 4 Department of Emergency Medicine, St James’s Hospital, Dublin 8, Ireland; 5 Department of Epidemiology and Public Health, University College Cork, Western Rd, Cork, Ireland; 6 Department of Emergency Medicine, Cork University Hospital, Cork, Ireland; 7 Institute for Clinical Evaluative Sciences, G1 06, 2075 Bayview Avenue, Toronto, ON M4N 3M5 Canada; 8 Division of Population Health Sciences, Royal College of Surgeons in Ireland, Dublin, Ireland; 9 Department of Emergency Medicine, Naas Hospital, Naas, Co, Kildare, Ireland; 10 School of Medicine, University College Cork, Western Rd, Cork, Ireland

**Keywords:** Longitudinal patient monitoring, Early warning score, Emergency department, Socio-technical systems, Participatory AR, Process and outcome evaluation

## Abstract

**Background:**

Early detection of patient deterioration is a key element of patient safety as it allows timely clinical intervention and potential rescue, thus reducing the risks of serious patient safety incidents. Longitudinal patient monitoring systems have been widely recommended for use to detect clinical deterioration. However, there is conflicting evidence on whether they improve patient outcomes. This may in part be related to variation in the rigour with which they are implemented and evaluated. This study aims to evaluate the implementation and effectiveness of a longitudinal patient monitoring system designed for adult patients in the unique environment of the Emergency Department (ED).

**Methods:**

A novel participatory action research (PAR) approach is taken where socio-technical systems (STS) theory and analysis informs the implementation through the improvement methodology of ‘Plan Do Study Act’ (PDSA) cycles. We hypothesise that conducting an STS analysis of the ED before beginning the PDSA cycles will provide for a much richer understanding of the current situation and possible challenges to implementing the ED-specific longitudinal patient monitoring system. This methodology will enable both a process and an outcome evaluation of implementing the ED-specific longitudinal patient monitoring system. Process evaluations can help distinguish between interventions that have inherent faults and those that are badly executed.

**Discussion:**

Over 1.2 million patients attend EDs annually in Ireland; the successful implementation of an ED-specific longitudinal patient monitoring system has the potential to affect the care of a significant number of such patients. To the best of our knowledge, this is the first study combining PAR, STS and multiple PDSA cycles to evaluate the implementation of an ED-specific longitudinal patient monitoring system and to determine (through process and outcome evaluation) whether this system can significantly improve patient outcomes by early detection and appropriate intervention for patients at risk of clinical deterioration.

## Background

### Introduction

The early recognition of the patient whose clinical condition is deteriorating is a key patient safety strategy, enabling timely clinical intervention to prevent serious adverse incidents for patients [1–4]. Longitudinal patient monitoring systems, for example, the Early Warning Scores (EWS) (NEWS in the UK and Ireland) and the Maternity Early Warning System (MEWS), are recommended to detect the deteriorating patient in many countries [5–9] despite conflicting evidence as to their success at improving patient outcomes [10–13]. Challenges to the successful implementation and evaluation of EWS include failure to heed the socio-cultural and organisational context [14, 15] and implementation in a ‘piecemeal’ manner without acknowledging the complexity of such an intervention [16]. This study is concerned with the implementation evaluation of a longitudinal patient monitoring system specifically designed for adult patients in the unique environment of the Emergency Department (ED) setting. This system is known as ED-ACE where ACE stands for Adult Clinical Escalation. A participatory action research (PAR) approach is taken where socio-technical systems (STS) theory and analysis informs the implementation and evaluation through the improvement methodology of multiple sequential ‘Plan Do Study Act’ (PDSA) cycles.

Lack of understanding of the complexity of forces acting both within and on healthcare systems has led to many failures in attempting to improve patient outcomes [17, 18]. The many potential advantages to healthcare of applying STS theory described by Carayon et al. ([19] p.3) as “adopting a systems approach aimed at identifying multiple system elements, their interactions and their impact on the quality of care, as well as understanding the key adaptive role of people in the system” have been highlighted [19–27]. The term STS was coined by Trist and colleagues in the Tavistock Institute in London in the 1950s and later taken up by Klein to recognise the interaction between technical and social factors in organisations [28–30]. When trying to change a system STS would stress the need to consider the technical and social factors and the impact of the change on other aspects of the system [31, 32].

This study applies STS theory and analysis for the first time to the implementation and evaluation of an ED longitudinal patient monitoring system. STS analysis will be conducted to describe and understand the ED environment and to inform the implementation and evaluation of ED-ACE. This analysis will take place at three levels: process functionality; communication, information and knowledge flow; and the social system (social relations, team, trust and accountability) using an STS analysis framework that has been developed in aviation safety research [33–35]. STS allows us to analyse the transformation of information into knowledge and the sharing of that knowledge and therefore can be applied to systems where there is currently a reliance on paper-based charts and whiteboards as in many EDs [36]. The ED-ACE being implemented and evaluated in this study is paper-based. STS also allows us to explore the team level interactions and the trust between team members as this is essential to ED functioning [37]. Finally, STS analysis allows us to focus on the process at the level at which it is relevant to the proposed implementation of ED-ACE. The ED patient pathway has already been mapped out in Ireland as part of the National Emergency Medicine Programme (EMP) [38] and the analysis will build on this. This more thorough understanding of the current system will similarly inform the evaluation framework.

While STS theory and analysis will inform this study the overarching implementation approach will be that of PAR as there is evidence that even very well-resourced change initiatives are ineffective if healthcare staff are not centrally involved in the design of the intervention [39]. PAR focuses on the effects of the researchers direct actions within a participatory community. The actions have a set goal of addressing an identified problem in the workplace and improving the performance quality of the community or area of concern [40–43].

Taylor et al. ([44] p.1) argue that PDSA cycles can help deliver improvements in healthcare that require the alteration of processes within “complex social systems that change over time in predictable and unpredictable ways”. They note that “in comparison to more traditional healthcare research methods (such as randomised controlled trials in which the intervention is determined in advance and there is an attempt to eliminate or control), the PDSA cycle presents an externally valid and pragmatic scientific method for testing changes in complex systems” ([44] p.2). However they do argue that there needs to be a theoretical framework against which the implementation of PDSAs is evaluated. They argue that this evaluation framework, which will be employed here, should include five key steps; “use of iterative cycles, initial small-scale testing, prediction-based testing of change, use of data over time and documentation” ([44] p.6).

### The research team and the emergency department

The Research Collaborative in Quality and Patient Safety (RCQPS), under which this project is funded, is a collaboration involving the Health Research Board, Ireland, the Health Service Executive (HSE), Ireland and the Royal College of Physicians of Ireland. The HSE is the statutory provider responsible for all the public health and social services in hospitals and in the community in the Republic of Ireland. The aim of the initiative is to bring researchers and clinicians together to generate research evidence in response to current quality and patient safety concerns. Research questions were developed and prioritised by the HSE’s National Clinical Programmes. Then, clinicians from the National Clinical Programmes were matched and partnered with expert researchers from a broad range of disciplines and backgrounds, including Health Systems, Epidemiology and Public Health, Psychology and Human Factors. The clinicians and researchers worked together to develop a research design and submit their proposals. The research methodology presented here reflects the multi-disciplinary nature of the team.

### The development of an ED-specific longitudinal patient monitoring system

The length of time patients spend in EDs, as measured by patient experience times (PETs), ED crowding and access block, represent one such concern from a patient safety and quality perspective in Ireland and many other countries. PETs of up to 115 h for discharge from the ED, and up to 140 h for admission to a hospital ward bed, have been reported [45]. In 2012, the Health Information and Quality Authority (HIQA), the statutory government-funded agency which monitors the safety and quality of health and social services in Ireland, recommended implementing “An emergency department specific system of physiological monitoring and triggered responses comparable to the National EWS” ([45] p.17). Roland and Coats [46] and Griffiths and Kidney [7] argue that there is undoubtedly a need for an ED-specific track and trigger system, but simply using an inpatient-derived model is potentially flawed because the external validity may be limited. The ED is a unique environment of uncontrollable patient volume and brief clinical encounters of variable acuity [47]. For the most part, ED patients are likely to be unknown to ED clinical staff and their illnesses are undifferentiated. They usually have to be managed with limited clinical information, through small windows of time and focus. Additionally, because of the acuity and the undifferentiated nature of their presenting clinical conditions, ED patients can have a relatively high potential for physiological instability requiring critical-care type interventions. With this in mind the National Emergency Medicine Programme (EMP), the HSE’s National Clinical Programme aimed at improving the safety and quality of care for ED patients in Ireland, developed and piloted ED-ACE through working with a multidisciplinary ED clinician group across six sites that involved over 175 ED staff and 2000 patient care episodes over an 18-month period [48].

**Fig. 1:**
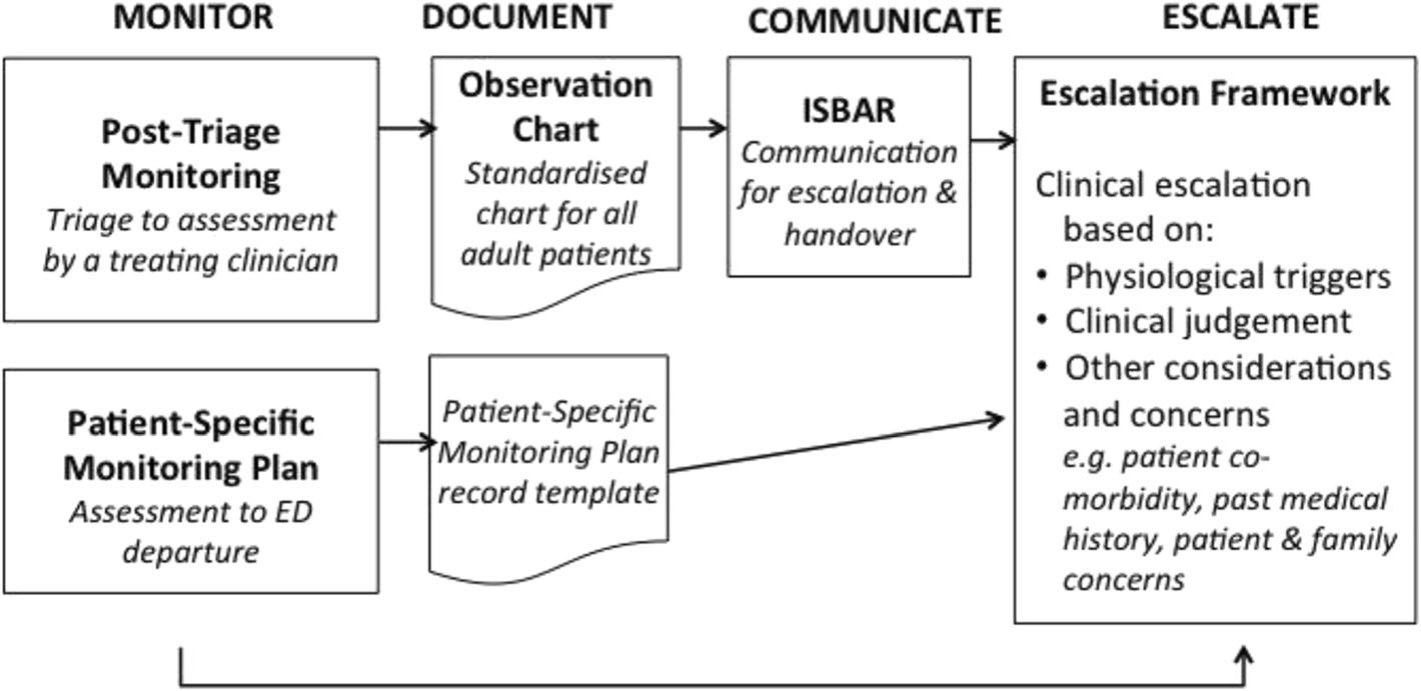
How the 5 components of ED-ACE link together

The development and piloting of the ED-specific longitudinal patient monitoring system, which is described in Coughlan et al. [48], was influenced by the requirement to optimise its alignment to the greatest degree possible with the existing NEWS used for inpatients while prioritising the unique physiological monitoring needs of the ED patient cohort. Thus ED-ACE comprises 5 clinical tools to facilitate early recognition and response/escalation to physiological deterioration in adult patients in the ED setting. The 5 clinical tools are: a longitudinal patient monitoring chart; a standardised approach to the monitoring and reassessment of patients after triage until such time as they are assessed by an ED doctor or Advanced Nurse Practitioner (ANP); an ISBAR tool for inter-professional communication relating to clinical escalation; a template for prescribing a patient-specific monitoring plan to be utilised by doctors and ANPs to guide patient monitoring from the time the patient is assessed until when they leave the ED; and a protocol for clinical escalation prompted by physiological triggers and clinical concern. Figure 1 details how the different elements of the ED-ACE tool relate to each other.

The aim of study is to evaluate the implementation and effectiveness of ED-ACE in assisting staff in the early recognition of patients whose clinical condition is deteriorating, thereby enabling timely clinical intervention to prevent serious adverse incidents for patients and to understand and/or explain what influences implementation outcomes relevant to ED-ACE. Implementation and evaluation of ED-ACE will take place in the ED of an Irish 1000-bedded academic teaching hospital considered representative of many of the EDs of other similar tertiary hospitals nationally.

## Methods

### Phase 1: assess the current situation or the ‘AS IS’ system

STS analysis of the current ‘AS IS’ ED system at the study site will take place at three levels: process functionality; communication, information and knowledge flow; and social relations. This analysis is guided by a framework that has been developed in aviation safety and is known as the ‘System Change and Operations Evaluation’ or ‘SCOPE’ framework [33–35]. The components of the SCOPE analysis of the current system are outlined in Table 1.

**Table 1:** SCOPE STS analysis to be applied to the current ED system

System component	Description	Analysis	Methods
Process functionality	The sequence of steps involved in the patient pathway through the ED where this relates to ED-ACE. The basic organising principle of STS is sequence, not necessarily in a simple linear fashion, but encompassing parallel activities, feedback loops and iterations. Resources (people/information/material) are transformed through tasks into outcomes that have value.	This will build on the EMP process map of the patient pathway and delve deeper into where the ED-ACE will be used. Critical points along the ED care pathway and key dependencies at these points will be identified. STS analysis will be carried out on the nature of these dependencies and in particular on the nature of any uncertainty being managed. In particular we will explore the supply of resources (people, information and material), their transformation through tasks and the co-ordination or management of associated dependencies.	The post-doctoral researcher will undergo a period of immersion in the day-to-day working of the ED shadowing staff members, tracking patients through the system, observations (e.g., of communication and information sharing at board rounds), corridor conversations and informal meetings, taking of ethnographic field notes. How work actually happens (the informal system) will be compared to the existing EMP map of how work should happen (the formal system).
The flow of information and the sharing of knowledge	Shared information, knowledge and understanding drive co-ordination, and other intentional acts. This knowledge and understanding is not necessarily explicit, expressed and formalised; it provides the practical ‘know-how’ that justifies and supports action and anticipates the consequences of action. Such actions and interactions (of people and technology) create facts/data that are then used to re-shape our understanding of the system.	Current documentation in use will be examined including patient charts, patient monitoring and recording, workflow charts, staffing quotas, performance reports including patient flow, quality reports and safety reports. The current key performance indicators (KPIs) and safety performance indicators (SPIs) will be examined, how are these measured, how are they displayed, fed back to staff, communicated to patients, how is the data that is currently gathered used for system improvement, what are the current drivers of system performance.	The post-doctoral researcher will attend monthly clinical risk meetings and hospital seminars on patient care flow, analysis of EMP-relevant KPIs, SPIs and flow charts, exploration of data captured on the hospitals patient information monitoring system (PIMS) reports (e.g., times along patient care pathway), ED documentation analysis including patient charts.
The social system	Work happens in organisations because people facilitate it happening through a series of social relations, team structures, trust relationships and accountability.	An analysis of the social system will be formed through observations of activity in ED and carrying out interviews with key stakeholders in the hospital’s emergency care system including triage nurses, nurses, clinical nurse managers, non-consultant hospital doctors, registrars and consultants in emergency medicine, administration, management, risk management and patient representation.	Interviews will be carried out with a sample of nurses and clinicians and administrative staff. The exact sample size will be determined by the data gathered – once data saturation has been reached the interviews will stop. Purposive sampling will be employed for the interviews. Coding and thematic analysis will be carried out on the data by two researchers. The coding will be supported by NVivo software package.

Following this activity a PAR group consisting of key ED stakeholders including triage nurses, other ED staff nurses, clinical nurse managers, non-consultant hospital doctors, consultants in emergency medicine, administration, management and patient representation will be developed. Working with the PAR group and with this richer picture of the ‘AS IS’ system an understanding of the current barriers and facilitators to implementing ED-ACE will be identified. From this the topics that need to be addressed during the different stages of implementation, if the risk of change failure is to be managed successfully, will be identified.

### Phase 2: participatory design of the ideal future/‘TO BE’ system including developing evaluation measures

**Fig. 2:**
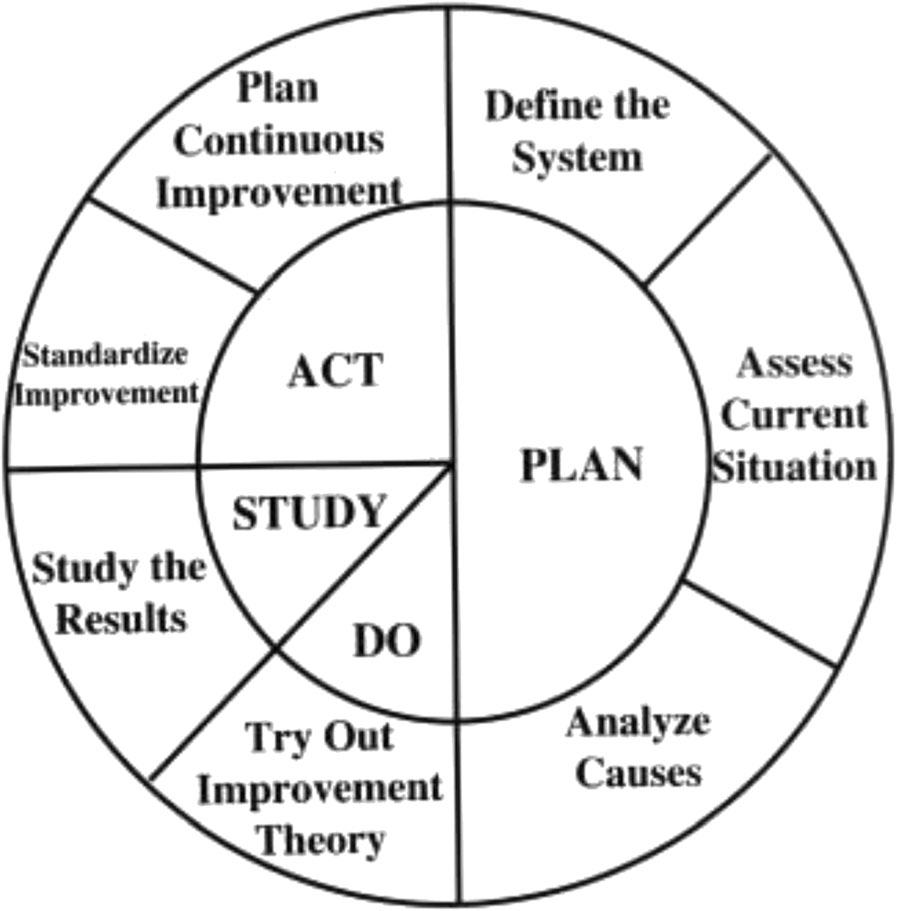
Plan Do Study Act Cycle [55]

In Phase 2 work will begin on designing how the current ED system needs to change to support the introduction of the ED-ACE. This involves identifying what needs to change if we are to move from the ‘AS IS’ to the ‘TO BE’ system where ED-ACE would be implemented. This movement will involve the use of PDSA cycles which the PAR group will design. The Institute for Healthcare Improvement (IHI) PDSA Worksheet for Testing Change will be used to help in this planning [49]. The four stages of the PDSA cycle are: Plan - the change to be tested or implemented; Do - carry out the test or change; Study - data before and after the change and reflect on what was learned; Act - plan the next change cycle or full implementation (Fig. 2).

Phase 2 involves the ‘Plan’ part of the PDSA cycle. For example, the planning stage will answer questions such as: In what ED shift will the first PDSA cycle take place?; How many staff will be involved in the first PDSA cycle and from which specific areas of the ED?; How will ED-ACE be used in conjunction with the current ED triage system?; What training will be given to staff?. This phase also involves exploring what will the process and outcome measures be and how will they be captured.

#### Study evaluation measures

There are currently no published scientifically valid process or outcome measures for measuring the effectiveness of implementing a longitudinal patient monitoring system in the ED setting. Therefore, as part of the ‘TO BE’ analysis, it is necessary to explore what are the ways in which successful implementation will be measured or what process and outcome measures will be employed. Oakley et al. ([50] p.413) recommend that process evaluations take place when implementing complex interventions as they “explore the implementation, receipt and setting of an intervention and help in the interpretation of the outcome results”. Process evaluations may include questionnaires, surveys, interviews, observations and field notes [50]. Process data will be analysed before outcome data to avoid bias in interpretation.

Choosing primary and secondary outcome measures for the implementation of a complex intervention in the ED setting such as ED-ACE is difficult. The ED does not function in isolation and during the patient journey through the emergency care system it contributes a small (but important) part of the care of a critically ill patient. Patient outcomes are often distant to direct ED clinical intervention. Therefore, a number of conceptual and practical criteria must be weighed before selecting such measures. These include the extent to which the outcome measure addresses both the benefits and potential harms of the intervention, the ease with which data about the outcome can be collected, the frequency of categorical events, and the relevance of the measure to the short and long-term goals of the intervention. There are five main types of measures that may be considered. First, mortality rates for patients attending the ED, either all-cause or disease-specific. These have the advantage of addressing clinically important outcomes for patients attending EDs, but have the disadvantage of being infrequent events, which would imply impractically large sample sizes. Second, are the unplanned critical events in the ED such as cardiac arrest, unexpected ICU admission or clinically important deterioration. These measures have the advantage of being conceptually proximal to ED care and the quality of ED patient monitoring but the disadvantage of being relatively infrequent. Third, timeliness measures such as ED waiting times for patients who experience clinically important deterioration. These measures are again clearly aligned with the quality of ED monitoring and likely to improve prognosis but have the disadvantage of being relatively difficult to measure. Fourth, adherence measures such as the proportion of patients for whom ED-ACE is properly adhered to by ED staff. This has the advantage of being a clear measure of the extent to which the intervention is properly implemented but the disadvantage of providing little information about the clinical benefits of the intervention to patients. Finally, the use of activity measures such as admission rates and the number of patients who are escalated to senior medical staff. These measures have the advantage of providing an insight into the potential stresses on the ED workforce and wider hospital environment of introducing the new monitoring tools, but again may not directly impact on patient health outcomes.

Thus, due consideration needs to be given to selecting measures that allow definitive and valid conclusions to be drawn from the study, while at the same time being meaningful and possible to track in the specific study setting. For this reason, we have decided to pool the expertise of the researchers, clinicians and other healthcare staff to develop a definitive set of measures. The methodology to achieve this is a two-stage process. Stage one will consist of an evaluation workshop whose purpose will be to develop suggestions for the evaluation process and outcome measures to be used. This workshop will take place with the PAR group whose membership is outlined above. The qualitative method of using paper ‘stickies’ to allow each person to generate as many suggestions for evaluation measures as they deem appropriate will be employed [51]. These suggestions will then be grouped into themes and any duplicates eliminated. The IHI framework of process, outcome and balancing measures will be used to structure the remaining evaluation measures [52]. We will divide process measures into those pertaining to treatment and those pertaining to implementation.

An electronic modified-Delphi study [53] will be carried out to reach consensus on an agreed set of evaluation measures that all staff involved and the project team will then agree are the best way of evaluating whether implementation of ED-ACE improves quality of care outcomes and patient safety [54]. For the Delphi a panel of experts will be chosen to include the research group, the Scientific Advisory Panel, the PAR group, other relevant stakeholders from the hospital (including the Risk Manager, ED consultants, registrars, advanced nurse paramedics and nurses), members of the EMP and the Emergency Medicine Nursing Interest Group, and Emergency Medicine and nursing leads in all the similar EDs throughout Ireland. Other methods commonly used to achieve consensus (e.g., a focus group) would not be feasible as the expert panel for this study will represent diverse geographical locations within Ireland that it would be impractical and costly to meet in person [53]. Strengths of the Delphi technique that make it suitable for our study include participant anonymity (to each other, though not the researcher), and the avoidance of group think or domination that might arise in a face-to-face discussion.

Thus, Delphi panel members will be asked to select measures relating to the following: (a) Treatment Process Measures – these measures examine the treatment process of patients in the ED and how that might be affected by ED-ACE; (b) Implementation process measures which will explore the implementation, the receipt and the setting of implementing ED-ACE and help in the interpretation of the outcome results. They can help distinguish between interventions that are inherently faulty and those that are badly delivered; (c) Outcome Measures – these measures should determine if ED-ACE results in improvement in patient outcomes; (d) Balancing measures look at a system from different directions/dimensions. They can help us answer questions such as, are changes designed to improve one part of the system by implementing ED-ACE causing new problems in other parts of the system? Statistical analysis will be informed by the results of the Delphi process and the selection of evaluation measures for investigation.

### Phase 3: the PDSA cycles

**Fig. 3:**
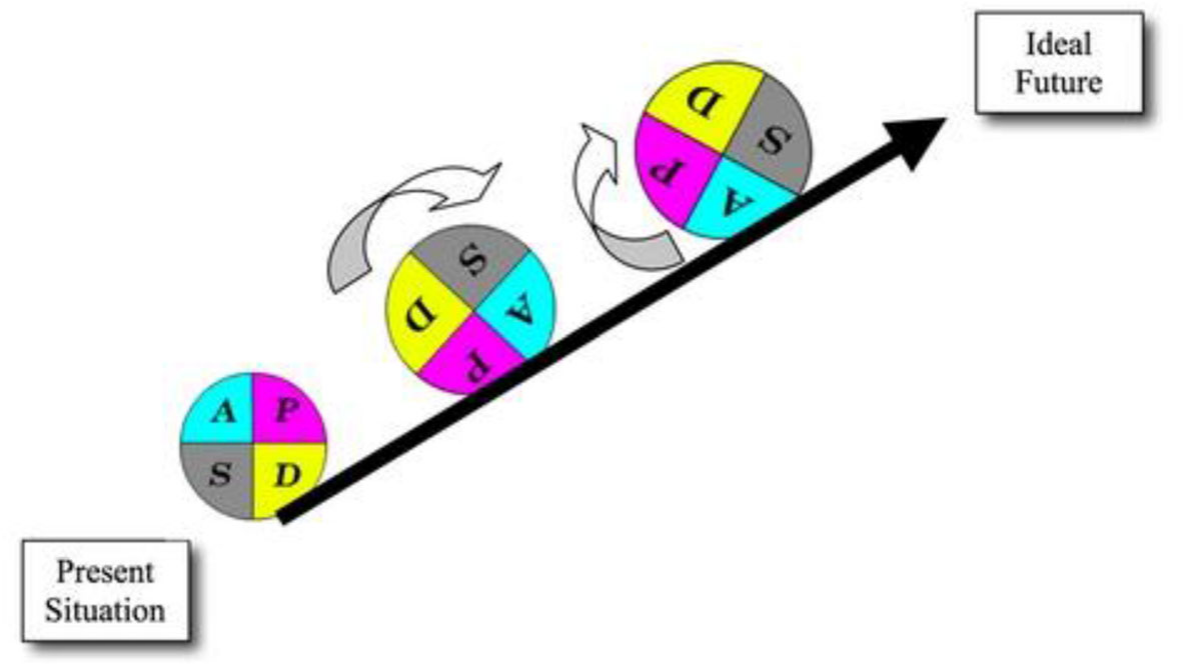
Multiple PDSA cycles [55]

PDSA cycles will be used to implement ED-ACE. The smallest PDSA is said to be ‘one patient, one time’. We plan to keep initial PDSAs small, but given the team nature of ED work, this may not be feasible with one ED staff member. The ‘DO’ stage will be documented with a particular focus on the challenges encountered and any unexpected outcomes. The project team will at each ‘STUDY’ stage of the PDSA cycle carry out a thorough analysis of the implementation to date, how it met with our predictions and refine and develop the next PDSA cycle based on this analysis. At the ‘ACT’ stage we will implement ED-ACE again, taking into account any findings from the ‘DO’ stage. Every effort will be made to continuously improve the ‘AS IS’ situation and move towards the ideal ‘TO BE’ future situation by employing the multiple sequential PDSA cycles within the overall PAR framework (Fig. 3).

At each STUDY stage the relevant agreed evaluation measures will be taken and studied and will inform subsequent stages of the PDSA and PDSA cycles. This as noted earlier will allow for a more thorough understanding of the challenges to be overcome in implementing and evaluating ED-ACE, in providing for active engagement and involvement of all staff involved, embedded learning and sustainable change.

### Study participants

Study participants will be drawn from the research team, the research project steering group, nursing and medical staff of the ED where the implementation will take place and membership at national level of specialist groups in Emergency Medicine. Table 2 below gives a detailed outline of study participants in the following four activities: STS analysis of the AS IS situation; PAR group; PDSA cycles; and the Delphi study to decide on evaluation measures.

**Table 2:** Study participants

Study Element	Study Participants
STS analysis of the AS IS situation	Interviews will be carried out with a sample of nurses and clinicians and administrative staff. The exact sample size will be determined by the data gathered – once data saturation has been reached the interviews will stop. Purposive sampling will be employed for the interviews. It is expected to interview at least 20 people.
PAR group	The research team members and key ED stakeholders including triage nurses, ED staff nurses, clinical nurse managers, non-consultant hospital doctors, consultants in emergency medicine, administration, management and patient representation. It is expected the PAR group will consist of 15–20 people.
PDSA cycles	As the PDSA cycles grow so too will the number of participants. The initial cycle will start small as per the PDSA approach and include 2 ED triage nurses.
Delphi study to decide on evaluation measures	The following people will be invited to join the Delphi panel: the research team and the research steering group committee; a selection of Consultants, REGs, ANPs from the ED who have not been involved in the research; all members of the national EMP and ENIG; the EM lead consultant and nursing leads in all the Model 4 EDs in the Ireland (comparable hospitals).

### Study status

The research team and PAR group have been established and the work of Phase 1 is complete, with the work of Phases 2 and 3 underway.

## Discussion

The position of the ED at the boundary between the hospital and its local population places it at the crossroads of multiple systems of care. In Ireland there are over 1.2 million annual ED attendances. Access blockages and long wait times in many EDs lead to ED crowding. This poses a risk to patient safety because deteriorating patients may go undetected during their prolonged stay in crowded EDs and are therefore at risk of developing serious adverse outcomes. Successful implementation of this new ED longitudinal patient monitoring system therefore has the potential of improving the quality of ED care and safety of a significant number of patients in the healthcare system. The ED longitudinal patient monitoring system being investigated aims to minimise clinical risk for ED patients through timely reassessment and appropriate clinical escalation for the duration of their ED-based care.

To determine the effectiveness of the implementation of ED-ACE, a novel mixed-methods approach is employed to evaluating its implementation, which to the best of our knowledge is the first study combining STS and PAR with multiple PDSA cycles. There are however a number of limitations that we are aware of.

While STS analysis has been used successfully in other industries and other areas of healthcare it has not been applied to the unique environment of the ED. The skill set of the research team and the active involvement of ED staff will help in applying STS to this new setting. Carrying out an STS analysis of the ED study site before beginning the first PDSA cycle provides the opportunity to gain an in-depth understanding of the current (‘AS IS’) situation and any potential challenges to implementing change. This more complete understanding and ability to identify challenges is an essential prerequisite for both the successful implementing and evaluation of change. STS analysis and a more thorough understanding of the current system will facilitate both a process and an outcome evaluation of implementing ED-ACE. Rychetnik et al. [cited in ([45] p.413)] note that process evaluations can help “distinguish between interventions that are inherently faulty (failure of intervention concept or theory) and those that are badly delivered (implementation failure)”.

Combining this with the PAR approach and involving the ED staff working in the current system will ensure that any challenges to achieving the ‘TO BE’ system will be identified and faced in a meaningful way. The strength of PAR is based on working in a collaborative and participatory manner with the staff in the ED study site and facilitating them to take ownership of the change process. May et al. ([43] p.6) highlight the benefits of this noting that PAR involves: “A continual reflective dialectic between theory and application of knowledge gained as a continuous research cycle. This reflective dialectic, involving ‘outsider’ professional university-based researchers, working collaboratively with ‘insider’ community-based researchers, opens traditional scientific knowledge to substantive incongruencies, inconsistencies and inaccuracies.” This can also be a weakness, however, as we are dependent on developing and fostering good relationships across the ED. This will hopefully be facilitated by having the post-doctoral researcher embedded in the ED, and working closely with the PI, who is a Consultant in Emergency Medicine at the study site.

PDSA cycles are widely used in healthcare but their utility to trial and test initiatives can be undermined by errors in their application, for example, being used in the incorrect order or not in cycles [44]. To counteract this, the evaluation framework proposed by Taylor et al. [44] will be used as a constant check to ensure that the PDSA cycles are correctly executed.

Thus a key strength of this study is its novel approach to evaluating the implementation and effectiveness of an escalation protocol. It adopts a systems perspective, aiming to develop an understanding of the environment in which the study is being conducted and combines process and outcome evaluations. The learning from this study will therefore not only provide an evaluation of ED-ACE, but will also contribute to the implementation science literature on complex interventions. The main limitation of the study is that it is taking place in one hospital only and therefore specific cultural factors in that hospital’s environment may have a considerable influence on the study findings.

Combining PAR, STS and PDSA may appear to be quite a labour intensive approach to implementing and evaluating the ED-ACE. However it is strongly believed that taking this innovative approach will allow us to document these possible cultural factors, and to develop both process and outcome evaluation measures. Carrying out both a process and outcome evaluation will help to overcome the limitations of previous studies and allow us to answer the question of whether the longitudinal patient monitoring systems significantly influences ED patient outcomes.

## Abbreviations

ANP: Advanced nurse practitioner
ED: Emergency department
ED-ACE: Emergency department adult clinical escalation
EMP: Emergency medicine programme
EWS: Early warning score
HIPE: Hospital in-patient enquiry
HIQA: Health information and quality authority
HSE: Health service executive
ICU: Intensive care unit
IHI: Institute for healthcare improvement
ISBAR: Identify situation background assessment recommendation
KPI: Key performance indicator
LPMS: Longitudinal patient monitoring system
MEWS: Maternity early warning score
PAR: Participatory action research
PDSA: Plan do study act
PET: Patient experience times
RCQPS: Research collaborative in quality and patient safety
SCOPE: System change and operations evaluation
SPI: Safety performance indicator
STS: Socio-technical systems
